# Side group dependent room temperature crystallization-induced phosphorescence of benzil based all organic phosphors†

**DOI:** 10.1039/d4ra00816b

**Published:** 2024-02-19

**Authors:** Sae Hui Lee, Marco S. Valverde Paredes, Paul M. Forster, Dong-Chan Lee

**Affiliations:** a Department of Chemistry and Biochemistry, University of Nevada Las Vegas 4505 S. Maryland Parkway, Box454003 Las Vegas Nevada 89154-4003 USA Dong-Chan.Lee@unlv.edu

## Abstract

In this work, we report alkoxy substituted benzil based all organic room temperature phosphors which showed crystallization induced phosphorescence (CIP). Nine title compounds were prepared with various alkyl lengths (OC_*n*_H_2*n*+1_: *n* = 8–16) and the effect of alkyl side group length on the phosphorescence performance was investigated, as compared to *p*-anisil. It was found that both phosphorescence quantum yield and lifetime increased concomitantly as the alkyl length increased up to nonyloxy (BZL-OC9). Further increase in the carbon number caused the phosphorescence performance to deteriorate due to greater conformational freedom of the side groups. An incredible quantum yield of 70% was achieved for BZL-OC9. A promising finding is that the increased quantum yield was accompanied by the increase in the lifetime relative to *p*-anisil, which has been historically challenging. Single crystallography coupled with UV-Vis spectroscopy revealed that a higher level of intermolecular π–π interactions was observed from *p*-anisil while more alkyl interactions with less intermolecular π-orbital overlap were found for BZL-OC8. As a result, molecular rigidification with less phosphorescence quenching was achieved for BZL-OC8 leading to enhanced performance. A precipitation study on a dichloromethane solution as a function of the content of MeOH (poor solvent) proved that the emission of the BZL-OC*n* system is truly aggregation-induced. The current work demonstrates that strategic side group engineering could be a promising approach to developing high-performance all organic phosphors as well as improving the properties of existing phosphors.

## Abstract 2

A systematic investigation on the effect of alkyl length on the phosphorescence performances of all organic room-temperature alkoxy substituted benzil phosphors.

## Graphical abstract

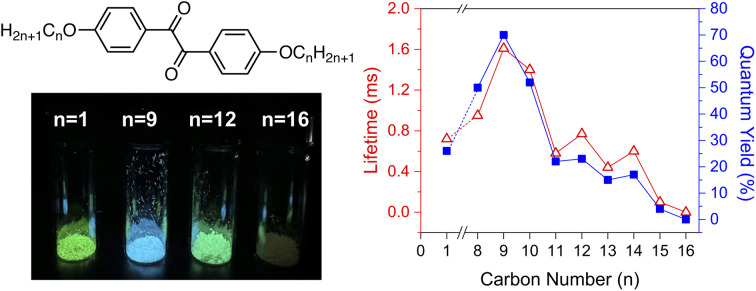

## Introduction

Organic room temperature phosphorescent materials have received a great deal of attention in recent years owing to their potential applications in organic light emitting diodes (OLED), organic photovoltaic devices (OPV), anti-counterfeiting technologies, sensors, and chemical and bio-imaging.^1^

Achieving phosphorescence at room temperature for metal-free all organic compounds is quite challenging due to the spin-forbidden nature of the triplet state causing inefficient spin–orbit coupling. In addition, triplet excitons experience thermal deactivations from various intramolecular motions and the quenching process by atmospheric oxygen and moisture.^2^ Therefore, metal-containing inorganic and organometallic compounds are still commonly utilized for room temperature phosphorescence (RTP).^3,4^ However, due to the potential benefits purely organic RTP systems have over metal-containing counterparts, such as cost-effectiveness, biocompatibility, low toxicity, and flexibility in synthesis, continuous efforts have been put into the development of efficient all organic phosphors.^1,5–14^

To realize organic RTP, efficient intersystem crossing (ISC) is necessary and molecular motion needs to be suppressed to prevent nonradiative thermal dissipation of excited energy. To promote ISC, a common approach is incorporating spin–orbit coupling (SOC) units such as heteroatoms or halogens.^15–19^ To prevent nonradiative relaxation caused by molecular motion, crystallization-induced phosphorescence (CIP)^1,5–10,20^ has emerged as a promising approach. CIP molecules typically do not show any phosphorescence at room temperature in solution, however, they exhibit phosphorescence as they crystallize. Tight molecular packing not only prevents oxygen from penetrating but freezes molecular motion as well, turning phosphorescence on at room temperature. Various systems have been developed based on organoboron complexes,^21–23^ persulfurated benzene,^24,25^ benzophenone derivatives,^20,26–28^ benzil derivatives,^29,30^ tellurophenes,^31,32^ 4,6-diphenyl-2-carbazolyl-1,3,5-triazine,^33^ heavy atom interactions^34,35^ and halogen-bonding^36–39^ based crystallization, *etc.* Among them, benzophenone derivatives have been the most actively studied systems.

Among the two parameters that account for the performance of RTP, lifetime and quantum yield, researchers have focused majorly on increasing lifetime, and impressive accomplishments have been made through molecular arrangement manipulation or new phosphor design. Extremely long lifetime has been achieved by stabilizing triplet states (*T*_n_) *via* H-aggregation.^33^ Also, increasing π-surface to promote ^1^(n,π*) to ^3^(π,π*) transition to populate further forbidden transition from ^3^(π,π*) to ^1^π^2^ has shown to increase lifetime.^26^

However, improving low quantum yield (<∼10%) of the RTP systems has been rather lagged.^8^ It is largely due to the forbidden nature of the radiative transition. When the triplet excited state is more populated to increase the lifetime, quantum yield suffers the adverse effect. Therefore, improving quantum yield together with lifetime has been quite a challenging task.

In CIP, the intrinsic maximum values for the lifetime and quantum yield of a given phosphor could only be achieved *via* the optimum molecular packing mode.^40^ Xie *et al.* have demonstrated that methyl and methoxy side groups can deteriorate the phosphorescence performance of carbazole-derivatized benzophenone as a result of loose and no face-to-face molecular packing.^41^ Conversely, with appropriate side groups that can assist an optimum molecular packing mode, both lifetime and quantum yield could improve simultaneously. However, there has not yet been any systematic study that could reveal the role of side groups. Therefore, in this work, we investigate the effect of alkyl side group length on the assembly of benzil phosphor and the entailing photophysical properties. Benzil was chosen as it is a relatively unexplored phosphor although it has shown a promising ISC and non-planar geometry^42,43^ that could avoid aggregation-caused quenching (ACQ).^44,45^

The CIP properties of benzil have been reported previously with only available lifetime data of 142 μs.^29^ Further detailed measurement from our research group found that benzil's quantum yield was only 2%. Very interestingly, *p*-anisil showed remarkable improvement in both lifetime and quantum yield when compared to benzil with the presence of methoxy substituent. The lifetime of *p*-anisil was found to be 0.72 ms which was *ca.* 5 times longer than that of benzil. The quantum yield of *p*-anisil was 26%, an even more remarkable increase from that of benzil. This result motivated us to believe that proper side group engineering could improve the CIP properties of a given phosphor. Therefore, in this work, we prepared nine alkoxy-substituted benzil with varying lengths. The overarching goal of this work is to demonstrate our strategy to improve both lifetime and quantum yield by simple side group engineering that can improve molecular arrangement in the solid-state. Their phosphorescence properties were carefully investigated in crystal form. Interesting trends in both lifetime and quantum yield as a function of side group length were observed, which was discussed along with single crystallographic data of *p*-anisil and octyloxy-substituted benzil.

## Results and discussion

**Fig. 1:**
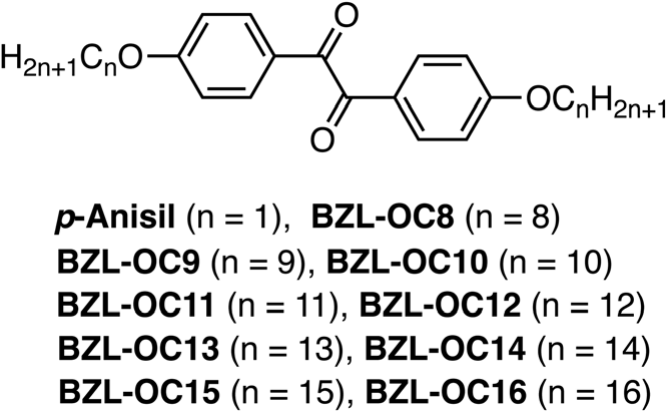
The molecular structures of title compounds with various alkyl lengths.

Fig. 1 shows the molecular structures of the target molecules with alkoxy substituents. Detailed synthetic methods for the target compounds are summarized in the ESI.† The target molecules were prepared by simple two-step reactions. The first step to generate BZL-OC*n* compounds was demethylation of *p*-anisil to produce 4,4′-dihydroxybenzil with 91% yield. Then, alkoxy substituents with different length were incorporated *via* an S_N_2 reaction (Williamson ether synthesis) with 66–91% yield.

**Fig. 2:**
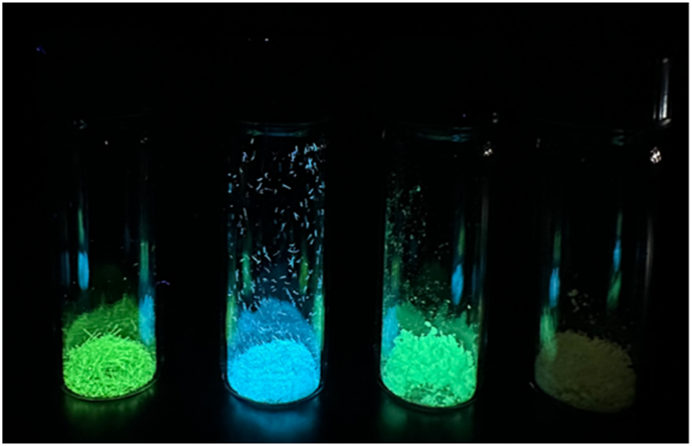
Emission of *p*-anisil, BZL-OC9, BZL-OC12, and BZL-OC16 (left to right) under a hand-held UV lamp (365 nm).

To investigate the photophysical properties of title compounds in the solid-state, crystalline form was prepared. The crystalline form was prepared by slowly evaporating diethyl ether solutions of title compounds at a concentration of 200 mM. All the title compounds exhibited bright luminescence under UV irradiation (365 nm) only in the solid-state except for BZL-OC16 which showed no detectable emission. Interestingly, *p*-anisil, BZL-OC12, and BZL-OC15 showed green emission color, while all other compounds showed blue emission under UV irradiation. Fig. 2 shows the pictures of green emissive (*p*-anisil and BZL-O12), blue emissive (BZL-OC9), and non-emissive (BZL-OC16) solids prepared from the slow evaporation of diethyl ether solutions.

**Fig. 3:**
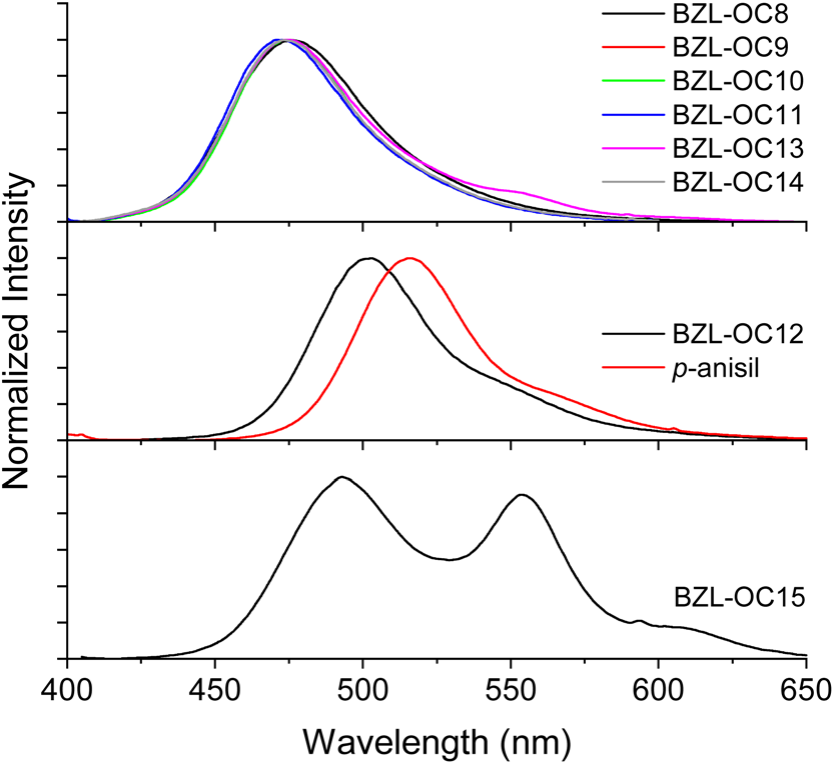
Prompt emission spectra of BZL-OC*n* molecules in crystal form. *λ*_ex_ from excitation spectra (see Table 1).

The prompt emission of title compounds in crystal form is shown in Fig. 3. All blue emissive compounds showed *λ*_em_ between 471 and 476 nm when excited at their respective *λ*_ex_ obtained from their excitation spectra (Table 1 and Fig. S1 in ESI†). The green emissive compound (BZL-OC12) showed a red-shifted *λ*_em_ at 503 nm when excited at 370 nm. Very interestingly, two distinctive *λ*_em_ at 493 and 553 nm were observed for BZL-OC15.

**Table 1:** *λ*_ex_, *λ*_em_, *τ*, and *Φ*_p_ values of BZL-OC*n* compounds prepared by the slow evaporation of diethyl ether solutions

	*λ*_ex_	*λ*_em_	*τ* (ms)	*Φ*_p_ (%)
*p*-Anisil	370	515	0.72	26
BZL-OC8	364	476	0.95	50
BZL-OC9	364	475	1.61	70
BZL-OC10	364	475	1.39	52
BZL-OC11	365	471	0.58	22
BZL-OC12	370	503	0.77	23
BZL-OC13	360	475	0.44	15
BZL-OC14	361	473	0.60	17
BZL-OC15	363	493, 553	0.10^a^, 0.52^b^	4
BZL-OC16^c^	NA	NA	0	0

a At 493 nm.

b At 553 nm.

c Negligible emission not being able to be measured.

**Fig. 4:**
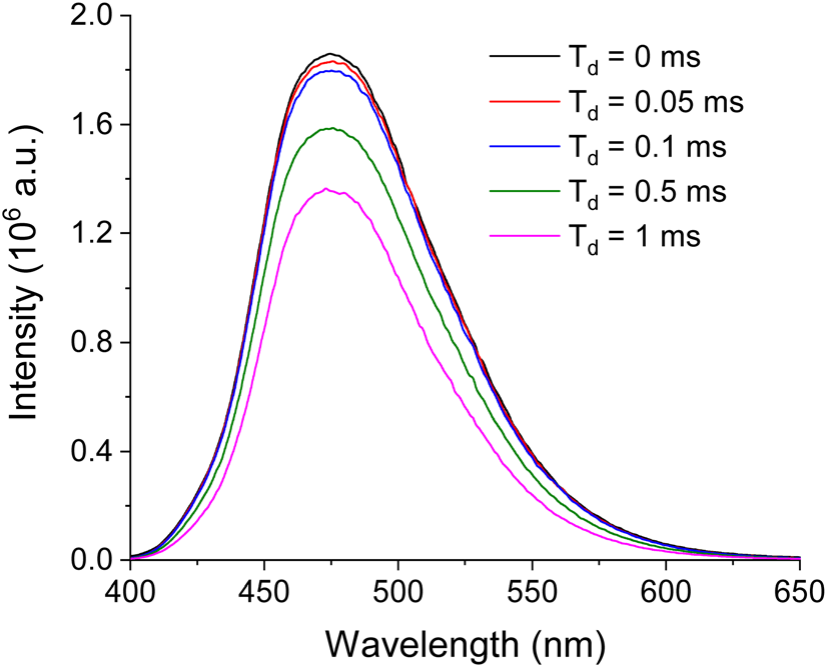
The emission spectra of BZL-OC9 at various delay time (*T*_d_).

To distinguish the origin of the emission, delay time experiments were performed. Since any emission with delay time (*T*_d_) longer than 0.1 ms excludes fluorescence contribution,^46^ comparing prompt emission and emission with *T*_d_ > 0.1 ms will verify whether the prompt emission is solely from phosphorescence. In this experiment, various *T*_d_'s up to 1 ms were applied. For all the samples, there was negligible spectral change including *λ*_em_ regardless of *T*_d_. BZL-OC9 as an example is shown in Fig. 4.

The only change with the increased *T*_d_ was a slight decrease in the emission intensity. From the prompt emission to *T*_d_ = 0.1 ms, the loss in the emission intensity was only 3%. For other compounds, the trend was consistent with that of BZL-OC9 and the maximum loss of intensity from *T*_d_ = 0 to 0.1 ms was less than 9%. This verifies that the origin of the title compound's emission is mostly from phosphorescence with a minor contribution of fluorescence.

**Fig. 5:**
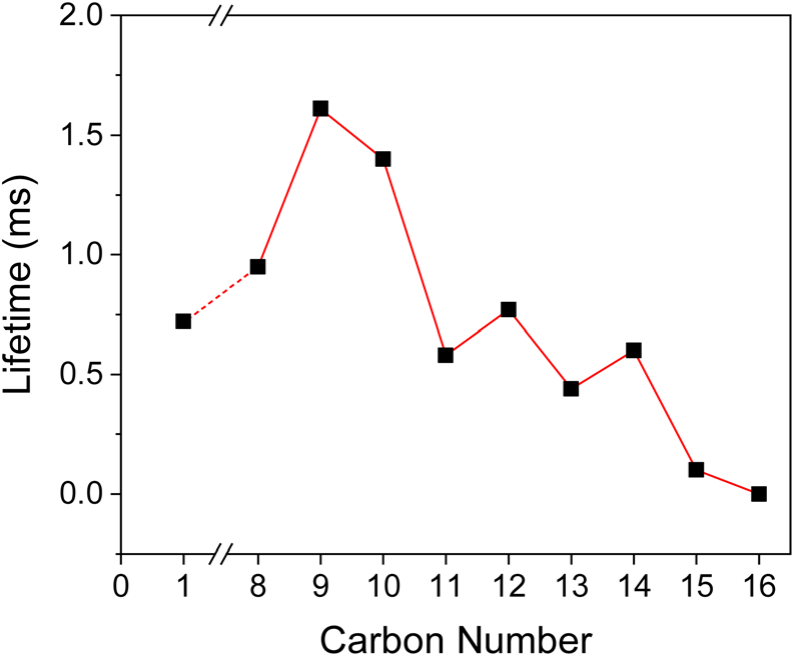
Phosphorescence lifetime as a function of carbon number in the alkyl side group in BZL-OC*n*. For BZL-OC15, the lifetime at 493 nm was used.

**Fig. 6:**
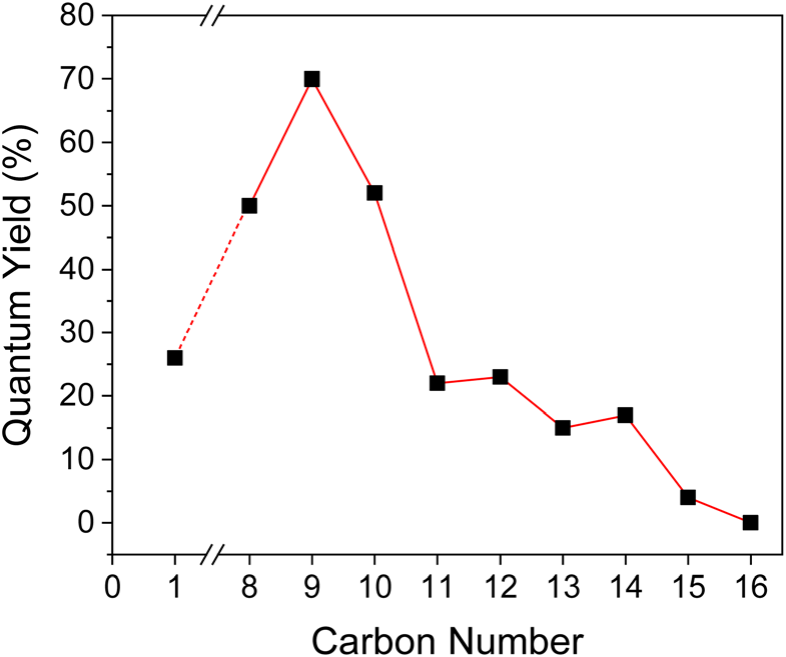
Phosphorescence quantum yield as a function of carbon number in the alkyl side group in BZL-OC*n*.

In order to evaluate the title compounds' phosphorescence performance, lifetime (*τ*) and quantum yield (*Φ*_p_) were measured which are summarized in Table 1, Fig. 5, and 6.

The *τ* of crystal was measured with time-resolved phosphorescence decay (Fig. S2, ESI†). As shown in Fig. 5, a continuous increase in *τ* was observed from *p*-anisil (0.72 ms) to BZL-OC8 (0.95 ms) to BZL-OC9 (1.61 ms). Then, slightly shorter *τ* was observed in BZL-OC10 (1.39 ms). For longer alkyl side groups, *τ* values were shortened below 1 ms: BZL-OC11 (0.58 ms), BZL-OC12 (0.77 ms), BZL-OC13 (0.44 ms), and BZL-OC14 (0.60 ms). In the case of BZL-OC15, two emission peaks were detected (Fig. 3), and distinctively different lifetime values were measured for each peak: 0.10 ms at 493 nm and 0.52 ms at 553 nm. Note that the longest *τ* (BZL-OC9) was *ca.* 2.24 times longer than that of *p*-anisil. The decrease in the lifetime when the number of carbons in the side group increased beyond 9 followed a zigzag style which is typical of an odd–even effect.^47^ The lifetimes of even carbon numbered alkoxy groups were longer than those of odd carbon numbers. A similar odd–even effect of alkyl chain on the lifetime of RTP has previously been observed from alkyl substituted phenothiazine 5,5-dioxide.^48^ For BZL-OC15, the lifetime at 493 nm was used for the plot. It should be noted the odd–even effect was still present when the lifetime at 553 nm was used.

Very interestingly, a similar trend was also observed from *Φ*_p_ of the crystal form. For BZL-OC8, -OC9, and -OC10, *Φ*_p_ values were over 50% (Fig. 6). The highest *Φ*_p_ of a remarkable 70% was observed from BZL-OC9. Considering that all organic phosphors with *Φ*_p_ value above 10% are hard to achieve, this finding is quite significant. In addition, it should be noted that even longer alkyl length compounds with relatively low *Φ*_p_ also had *Φ*_p_ over 10%, except for BZL-OC15 and -OC16. Similar to the lifetime, *Φ*_p_ decreased experiencing an odd–even effect from BZL-OC10 to -OC15. It is clear that the effect of the alkoxy side groups is consistent for both lifetime and *Φ*_p._

The results of *τ* and *Φ*_p_ measurements indicate that there seemed to be an optimum alkyl length to achieve the highest phosphorescence performance. Our hypothesis is that the side group ordering *via* van der Waals interactions between the alkyl chains effectively restricts the intramolecular motion (RIM) of benzil core and significantly lowers the rate of nonradiative decay. However, such an effect lasts only up to BZL-OC10. Presumably, the compounds with longer alkyl length are experiencing ineffective intermolecular aggregation due to the higher conformational freedom of longer alkyl chains, which results in more intramolecular motion with low phosphorescence performance. It should be noted that the remarkable improvement in *Φ*_p_ was achieved without deteriorating *τ*. Indeed, both parameters increased simultaneously for BZL-OC8, -OC9, and -OC10. This is quite an encouraging result which was accomplished through simple alteration of carbon numbers in the side groups.

**Fig. 7:**
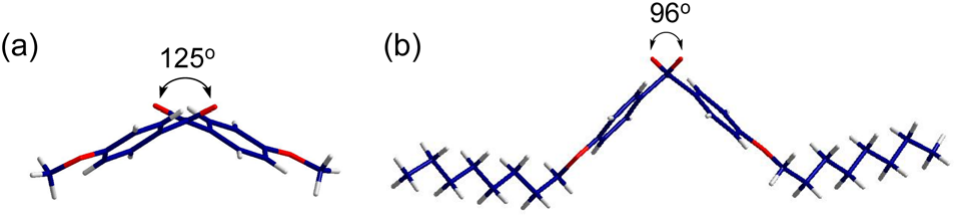
The dihedral angle of two carbonyl groups in (a) *p*-anisil and (b) BZL-OC8.

X-ray single crystallography was conducted to gain better insight into the molecular packing mode. Among all the samples, only *p*-anisil and BZL-OC8 yielded high quality single crystals with a size suitable for X-ray crystallography by slow evaporation of ether solutions. Details of the single crystal structure determinations are presented in the ESI.† The crystal structure of *p*-anisil has been reported previously.^30,43,49^ We are including our structure determination which converged to a better *R*_1_ value, although no other significant differences between the structures were found. Both crystals were found to be monoclinic with the unit cell parameters presented in Tables S1 and S2 (ESI).† The crystal unit cells of *p*-anisil and BZL-OC8 are shown in Fig. S3 (ESI).† Crystal *a*-, *b*-, and *c*-directions were along the molecular long axis, the molecular stacking direction, and the molecular short axis, respectively. BZL-OC8 had significantly increased unit cell in *c*-direction compared to the *p*-anisil due to the intercalation of octyloxy group between π-cores. Fig. 7 shows dihedral angles [C(O)–C–C–C(O)] of the two compounds around the central C–C bond between the two carbonyl groups. A considerable difference in dihedral angle was observed where *p*-anisil had a larger angle of 125°, and BZL-OC8 had a reduced angle of 96°. This shows the significant effect of alkyl chain length in the molecular assembly. Perhaps, the assembly of octyloxy group pushed both sides of BZL core to generate more kinked conformation with a more closed scissor-like shape in BZL-OC8.

**Fig. 8:**
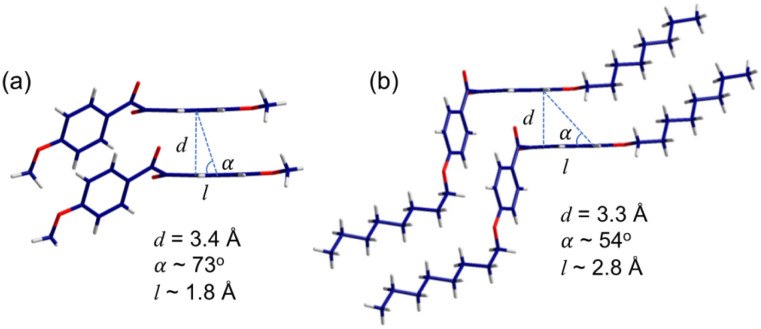
The π-core stacking mode of (a) *p*-anisil and (b) BZL-OC8.

Fig. 8a shows the crystallographic *b*-direction for *p*-anisil. The stacking direction shows inter π-core distance (*d*) of 3.4 Å which could enable effective π–π overlap between the molecules. For the solid-state emission, not only *d* is important but the degree of inter π-orbital overlap is crucial as well. The slip angle (*α*) between two neighboring molecules was calculated to be 73°, from which slip displacement distance (*l*) of 1.8 Å was also deduced. This slip displacement distance is shorter than the theoretical diagonal length of the benzene excluding hydrogens (2.7850 Å), which indicates a significant π–π overlap between the molecules in *p*-anisil. Fig. 8b shows the crystallographic *b*-direction for BZL-OC8. The inter π-core distance (*d*) was found to be 3.3 Å, which does not show much difference compared to that of *p*-anisil. However, a considerable difference was observed in the slip angle (*α*) and the slip displacement (*l*), which were found to be 54° and *ca.* 2.8 Å, respectively. This slip displacement distance is longer than the size of benzene, which indicates minimal inter π-overlap in BZL-OC8.

**Fig. 9:**
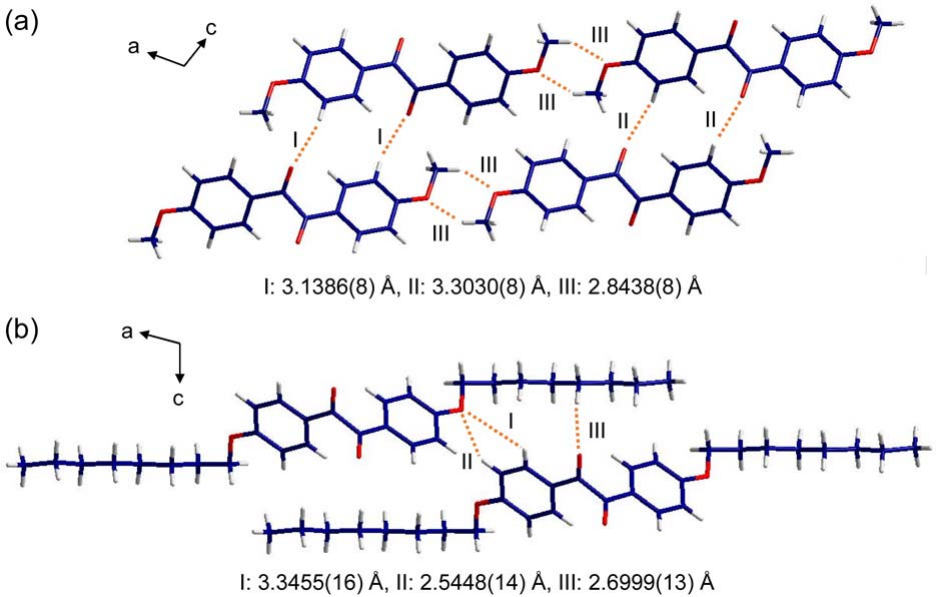
The crystallographic *a*- and *c*-direction of (a) *p*-anisil and (b) BZL-OC8.

More detailed information on the intermolecular interactions was revealed from the crystallographic *a*- and *c*-directions. For *p*-anisil, the interatom distances between O(1) and H(4) (I and II in Fig. 9a) were found to be 3.1386(8) Å and 3.3030(8) Å, respectively, which are within van der Waal radius. The two distances (I and II) were alternating along the *c*-direction. Also, the distance between O(2) in one molecule and H(12B) in the neighboring molecule (III in Fig. 9a) was 2.8438(8) Å, indicating that the neighboring methoxy groups are interacting with each other. In the case of BZL-OC8, as a consequence of the π-cores being slipped away from each other significantly, the interatom distances of O(1)–H(3) (3.3455(16) Å, I in Fig. 9b) and O(1)–H(4) (2.5448(14) Å, II in Fig. 9b) fell within the interaction range. In addition, the distance between O(2) and H(12A) was quite short (2.6999(13) Å, III in Fig. 9b).

The single crystal X-ray analysis verified significant differences in the intermolecular assembly modes and the resulting degree of π–π overlap when the alkyl side chain length was varied. It clearly shows that *p*-anisil with a shorter alkyl length has more π–π overlap than BZL-OC8.

**Fig. 10:**
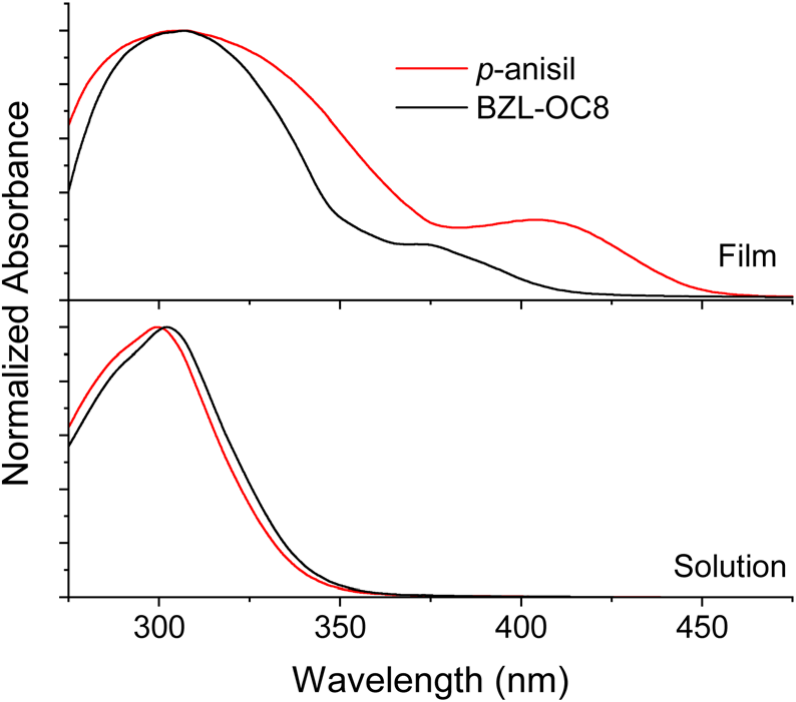
UV-Vis absorption spectra of *p*-anisil and BZL-OC8 in solution and cast film from DCM_._

Such a difference in the degree of π–π overlap was also evaluated by absorption behavior with UV-vis spectroscopy. Fig. 10 is the absorption spectra of *p*-anisil and BZL-OC8 as solutions and cast films prepared from dichloromethane (DCM) solutions. In solution, the absorption of both compounds was literally identical with *λ*_max_ at *ca.* 300 nm. However, the films showed new shoulders at longer wavelength. While a smaller shoulder appeared at *ca.* 371 nm for BZL-OC8, *p*-anisil showed a more prominent shoulder at 406 nm. The red-shifted shoulder of *p*-anisil is indicative of stronger π–π interactions, which is consistent with the observation from the single crystal data.

Taking all the data into consideration, we hypothesize that intermolecular π–π interactions between benzil cores may be the leading driving force for the assembly in *p*-anisil. However, in the case of BZL-OC8, the increased van der Waals interactions by the longer alkyl side group may have led the assembly. The *τ* and *Φ*_p_ data showed significant improvement in phosphorescence performance from BZL-OC8 compared to *p*-anisil with *ca.* 1.3 times increased τ and *ca.* 1.9 times increased *Φ*_p_. We presume that the side group-driven assembly of BZL-OC8 caused minimal inter π-orbital overlap while restricting the molecular motions, which resulted in enhanced phosphorescence by avoiding detrimental ACQ.

**Fig. 11:**
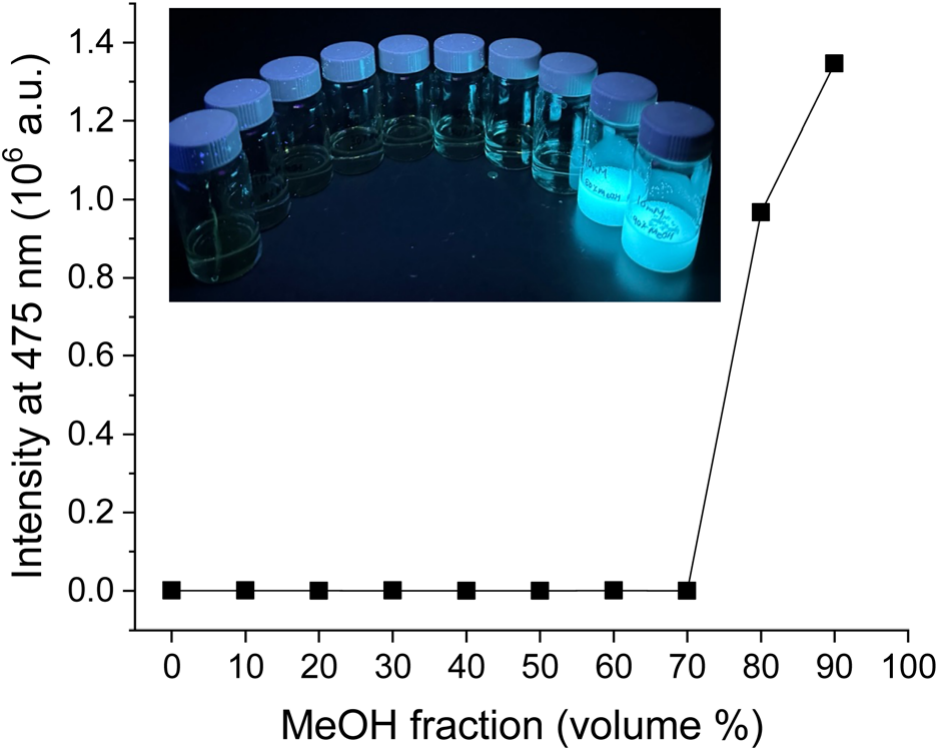
Emission study upon increasing the fraction of poor solvent (MeOH) in DCM solution of BZL-OC9 (10 mM). Inset: the picture of vials with increasing MeOH in DCM solution (from 0 to 90 vol% with 10% increment) under a hand-held UV lamp (365 nm).

To further confirm that the current system is CIP, we conducted a precipitation study on BZL-OC9. In this study, a poor solvent (MeOH) was added to a DCM solution of BZL-OC9 in 10 vol% increment up to 90%. For all the samples, the total concentration was kept at 10 mM. As shown in Fig. 11, visibly recognizable precipitates started to form from 80 vol% of MeOH. The emission was observed only when the precipitates were formed.

The precipitation study clearly demonstrates that the emission of BZL-OC*n* system is originated from aggregation.

## Conclusion

In the present work, we have successfully synthesized new purely organic room temperature phosphors based on BZL with varying alkyloxy side group length. An extraordinary *Φ*_p_ value reaching up to 70% was found from BZL-OC9. Considering the poor *Φ*_p_ values of most organic phosphors, BZL-OC9's *Φ*_p_ is a remarkable achievement. Additionally, such a high *Φ*_p_ was accompanied by an increased *τ*. The concomitant increase in *Φ*_p_ and *τ* is encouraging as improving the two parameters simultaneously has been quite challenging. We found that there is an optimum alkyl length to achieve the maximum phosphorescence performance. Enhanced phosphorescence performance compared to *p*-anisil was observed in BZL-OC8, -OC9 and -OC10, with -OC9 reaching the maximum. Further increase in alkyl length caused decreased phosphorescence performance in both lifetime and quantum yield showing an odd–even effect. This is perhaps due to the lack of efficient rigidification caused by increased conformational freedom of the longer side chains. The single crystal study on *p*-anisil and BZL-OC8 clearly revealed that there are two different modes of molecular assembly. More alkyl–alkyl interaction driven assembly was found in longer alkyloxy substituted BZL while π–π interaction led the molecular packing of shorter counterparts. Such assembly modes of longer alkyl-based BZL not only avoided strong intermolecular π-orbital overlap but helped rigidify the molecules as well, which suppressed ACQ.

The significance of the current study is that the dramatic improvement in *Φ*_p_ without sacrificing *τ* was accomplished by simple manipulation of alkyl side group length. How the π-core assembled influenced the solid-state photophysical properties drastically and such a change was possible by the change in a few carbon numbers in the side group. The findings in this work suggest that strategic side group engineering be a promising approach not only to develop high-performance all organic CIPs but to further improve the phosphorescence performances of existing organic phosphors as well.

## Conflicts of interest

There are no conflicts to declare.

## Supplementary Material

RA-014-D4RA00816B-s001

RA-014-D4RA00816B-s002
