# Supplementary material for: Side group dependent room temperature crystallization-induced phosphorescence of benzil based all organic phosphors
Source: RSC Adv. 2024 Feb 19;14(9):6285–91. doi: 10.1039/d4ra00816b (PMC10875412; doi:10.1039/d4ra00816b)
Supplement: RA-014-D4RA00816B-s001 [file RA-014-D4RA00816B-s001.pdf]

Electronic Supplementary Material (ESI) for RSC Advances.
This journal is © The Royal Society of Chemistry 2024

Side group dependent room temperature crystallization-induced phosphorescence of benzil based all organic phosphors

Sae Hui Lee, Parco S. Valverde Paredes, Paul M. Forster and Dong-Chan Lee

Instrumentation

Nuclear magnetic resonance (NMR) spectra were obtained with a Varian Gemini 400 MHz spectrometer at room temperature. Deuterated chloroform ($CDCl_3$) containing tetramethylsilane (TMS) as an internal standard was used as the solvent for both ^{1}H NMR and ^{13}C NMR. The mass spectra were collected with Advion Expression CMS under APCI mode. Absorption properties of the molecules were obtained with a Shimadzu UV-2600 UV-visible spectrophotometer for absorption. Emission and phosphorescence LT and QY, was measured with Horiba Fluorolog-3 spectrophotometer equipped with a Xenon lamp and integrating sphere. Single crystal X-ray diffraction (SCXRD) data were collected on a Bruker APEX II CCD instrument at 100 K with Mo−Kα radiation (λ = 0.71073 Å). A crystal was mounted under Paratone® on a glass fiber; data processing was performed using the Apex II suite software. Structural solution and refinements were completed using SHELXT program[1,2] and refinements were carried out in the OLEX2 program package.[3] Hydrogen atoms are placed at calculated positions and refined using riding models. Crystal parameters and refinement results are summarized in Table S1.

Synthetic Procedures

Below is the general synthesis procedure for BZL-OCn series comprises two step reactions. Demethylation of *p*-anisil (BZL-OC1) to produce 4,4'-dihydroxybenzil, followed by alkylation via SN2 reaction (Williamson ether synthesis) produced title compounds with reasonable yields. After deprotonation of 4,4'-dihydroxybenzil by potassium carbonate (K_2CO_3), alkoxide ions displace halide ion from alkyl halide in various alkyl lengths to produce BZL-OCn compounds. The final products were fully characterized with ^{1}H NMR, and ^{13}C NMR spectroscopy and mass spectrometry.

All Chemicals and solvents were purchased from commercial sources and used as received without further purification.

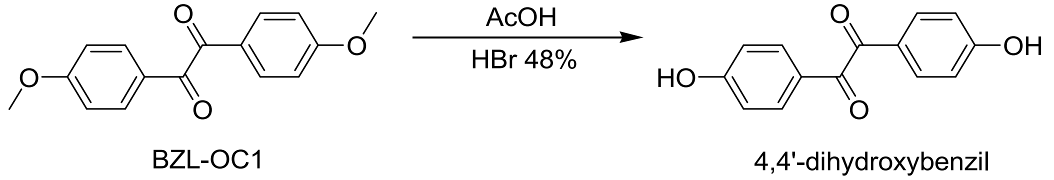

Scheme S1. Synthetic route for 4,4'-dihydroxybenzil.

4,4'-dihydroxybenzil was synthesized following the previously published procedures[4] with different reaction time. *p*-Anisil (3g, 11.1 mmol) was suspended in 48mL aqueous HBr (48%) and 22.5mL glacial acetic acid (AcOH). The reaction mixture refluxed for 73 hours under nitrogen atmosphere. After cooling to room temperature, it was poured into ice water and stirred for 10 minutes. Resulted precipitate was filtered, repeatedly washed with water and dried. Since product was analytically pure without further purification, additional purification method was omitted.

Final product had tan solid with 91% yield. ^{1}H NMR (400 MHz, DMSO) δ 10.79 (s, 2H), 7.72-7.70 (m, 4H), 6.91-6.89 (m, 4H).

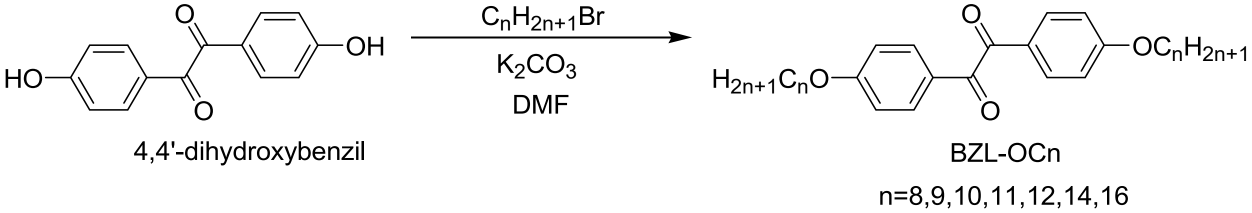

Scheme S2. Synthetic route for BZL-OCn derivatives.

The title BZL-OCn molecules have been previously reported,[5-10] and the synthesis of all of the BZL-OCn compounds followed previously published procedures with modified workup procedure and column chromatography condition. 4,4'-dihydroxybenzil (500mg, 1.71mmol), alkyl bromide (0.89mL, 5.13mmol), and potassium carbonate (K_2CO_3) (709mg, 5.13mmol) were dissolved in DMF (5mL) at 90 ˚C under nitrogen overnight. After cooling to room temperature, it was quenched by adding water. The resulted precipitate was filtered, and then dissolved in methylene chloride (CH_2Cl_2). The solution was dried over sodium sulfate (Na_2SO_4) and then the solvent was evaporated after filtration. The crude material was purified by silica gel column chromatography (eluent: n-hexane to ethyl acetate/n-hexane 2/98).

BZL-OC8: White solid (88% yield). ^{1}H NMR (400 MHz, $CDCl_3$) δ 7.93 (d, 4H, *J*= 8.8 Hz), 6.94 (d, 4H, *J*=8.8 Hz), 4.03 (t, 4H, *J*= 6.8 Hz), 1.80 (m, 4H), 1.47-1.28 (m, 20H), 0.89 (t, 6H, *J*=7.2 Hz). ^{13}C NMR ($CDCl_3$) δ 193.57, 164.49, 132.35, 126.08, 114.70, 68.47, 31.78, 29.28, 29.19, 29.90, 25.93, 22.63, 14.08. $[M+H]^+$: Calcd 467.3; Found 467.5.

BZL-OC9: White solid (89% yield). ^{1}H NMR (400 MHz, $CDCl_3$) δ 7.93 (d, 4H, *J*= 9.2 Hz), 6.94 (d, 4H, *J*=9.2 Hz), 4.03 (t, 4H, *J*= 5.60 Hz), 1.80 (m, 4H), 1.45-1.28 (m, 24H), 0.88 (t, 6H, *J*= 6.80 Hz). ^{13}C NMR ($CDCl_3$) δ 193.57, 164.49, 132.35, 126.08, 114.70, 68.46, 31.84, 29.48, 29.31, 29.22, 29.00, 25.92, 22.65, 14.09. $[M+H]^+$: Calcd 495.3; Found 495.5.

BZL-OC10: White solid (91% yield). ^{1}H NMR (400 MHz, $CDCl_3$) δ 7.93 (d, 4H, *J*= 9.2 Hz), 6.94 (d, 4H, *J*=8.8 Hz), 4.03 (t, 4H, *J*= 6.4 Hz), 1.80 (m, 4H), 1.47-1.27 (m, 28H), 0.88 (t, 6H, *J*=7.2 Hz). ^{13}C NMR ($CDCl_3$) δ 193.57, 164.49, 132.35, 126.08, 114.70, 68.46, 31.87, 29.52, 29.31, 29.29, 29.90, 25.92, 22.67, 14.10 (1 alkyl carbon peak not seen due to overlapping signals). $[M+H]^+$: Calcd 523.4; Found 523.6.

BZL-OC11: White solid (72% yield). ^{1}H NMR (400 MHz, $CDCl_3$) δ 7.93 (d, 4H, *J*= 8.8 Hz), 6.94 (d, 4H, *J*=9.2 Hz), 4.03 (t, 4H, *J*= 6.8 Hz), 1.80 (m, 4H), 1.47-1.27 (m, 32H), 0.88 (t, 6H, *J*= 7.6 Hz). ^{13}C NMR ($CDCl_3$) δ 19.357, 164.49, 132.35, 126.09, 114.70, 68.47, 31.90, 29.60, 29.57, 29.53, 29.32, 29.90, 25.93, 22.67, 14.11 (1 alkyl carbon peak not seen due to overlapping signals). $[M+H]^+$: Calcd 551.4; Found 551.6.

BZL-OC12: White solid (77% yield). ^{1}H NMR (400 MHz, $CDCl_3$) δ 7.93 (d, 4H, *J*= 8.8 Hz), 6.94 (d, 4H, *J*=8.8 Hz), 4.03 (t, 4H, *J*= 6.8 Hz), 1.80 (m, 4H), 1.47-1.26 (m, 36H), 0.88 (t, 6H, *J*= 7.2 Hz). ^{13}C NMR ($CDCl_3$) δ 193.56, 164.49, 132.35, 126.09, 114.69, 68.46, 31.91, 29.64, 29.62, 29.57, 29.53, 29.33, 29.32, 29.00, 25.93, 22.68, 14.11. $[M+H]^+$: Calcd 579.4; Found 579.5.

BZL-OC13: White solid (85% yield). ^{1}H NMR (400 MHz, $CDCl_3$) δ 7.93 (d, 4H, *J*= 8.8 Hz), 6.94 (d, 4H, *J*= 8.8 Hz), 4.03 (t, 4H, *J*= 6.8 Hz), 1.80 (m, 4H,), 1.48-1.26 (m, 40H), 0.88 (t, 6H, *J*= 6.8 Hz). ^{13}C NMR ($CDCl_3$) δ 193.58, 164.50, 132.37, 126.10, 114.71, 68.48, 31.92, 29.67, 29.64, 29.58, 29.54, 29.35, 29.33, 29.02, 25.93, 22.70, 14.12 (1 alkyl carbon peak not seen due to overlapping signals). $[M+H]^+$: Calcd 607.5; Found 607.6

BZL-OC14: White solid (66% yield). ^{1}H NMR (400 MHz, $CDCl_3$) δ 7.93 (d, 4H, *J*= 8.8 Hz), 6.94 (d, 4H, *J*=9.2 Hz), 4.03 (t, 4H, *J*= 6.4 Hz), 1.80 (m, 4H), 1.47-1.26 (m, 44H), 0.88 (t, 6H, *J*= 6.8 Hz). ^{13}C NMR ($CDCl_3$) δ 193.55, 164.49, 132.35, 126.09, 114.69, 68.47, 31.92, 29.68, 29.66, 29.65, 29.57, 29.54, 29.35, 29.32, 29.01, 25.93, 22.69, 14.11 (1 alkyl carbon peak not seen due to overlapping signals). $[M+H]^+$: Calcd 635.5; Found 635.5.

BZL-OC15: White solid (76% yield). ^{1}H NMR (400 MHz, $CDCl_3$) δ 7.93 (d, 4H, *J*= 8.8 Hz), 6.94 (d, 4H, *J*= 8.8 Hz), 4.03 (t, 4H, *J*= 6.8 Hz), 1.80 (m, 4H,), 1.47-1.26 (m, 48H), 0.88 (t, 6H, *J*= 6.8 Hz). ^{13}C NMR ($CDCl_3$) δ 193.58, 164.50, 132.37, 126.10, 114.71, 68.48, 31.93, 29.70, 29.69, 29.67, 29.66, 29.58, 29.54, 29.37, 29.33, 29.02, 25.93, 22.70, 14.12 (1 alkyl carbon peak not seen due to overlapping signals). $[M+H]^+$: Calcd 663.5; Found 663.6

BZL-OC16: White solid (82% yield). ^{1}H NMR (400 MHz, $CDCl_3$) δ 7.93 (d, 4H, *J*= 8.8 Hz), 6.94 (d, 4H, *J*=9.2 Hz), 4.03 (t, 4H, *J*= 6.6 Hz), 1.80 (m, 4H), 1.45-1.26 (m, 52H), 0.88 (t, 6H, *J*= 6.8 Hz). ^{13}C NMR ($CDCl_3$) δ 193.54, 164.47, 132.33, 126.07, 114.68, 68.46, 31.90, 29.67, 29.65, 29.63, 29.56, 29.51, 29.34, 29.30, 28.99, 25.91, 22.67, 14.10. $[M+H]^+$: Calcd 691.6; Found 691.5.

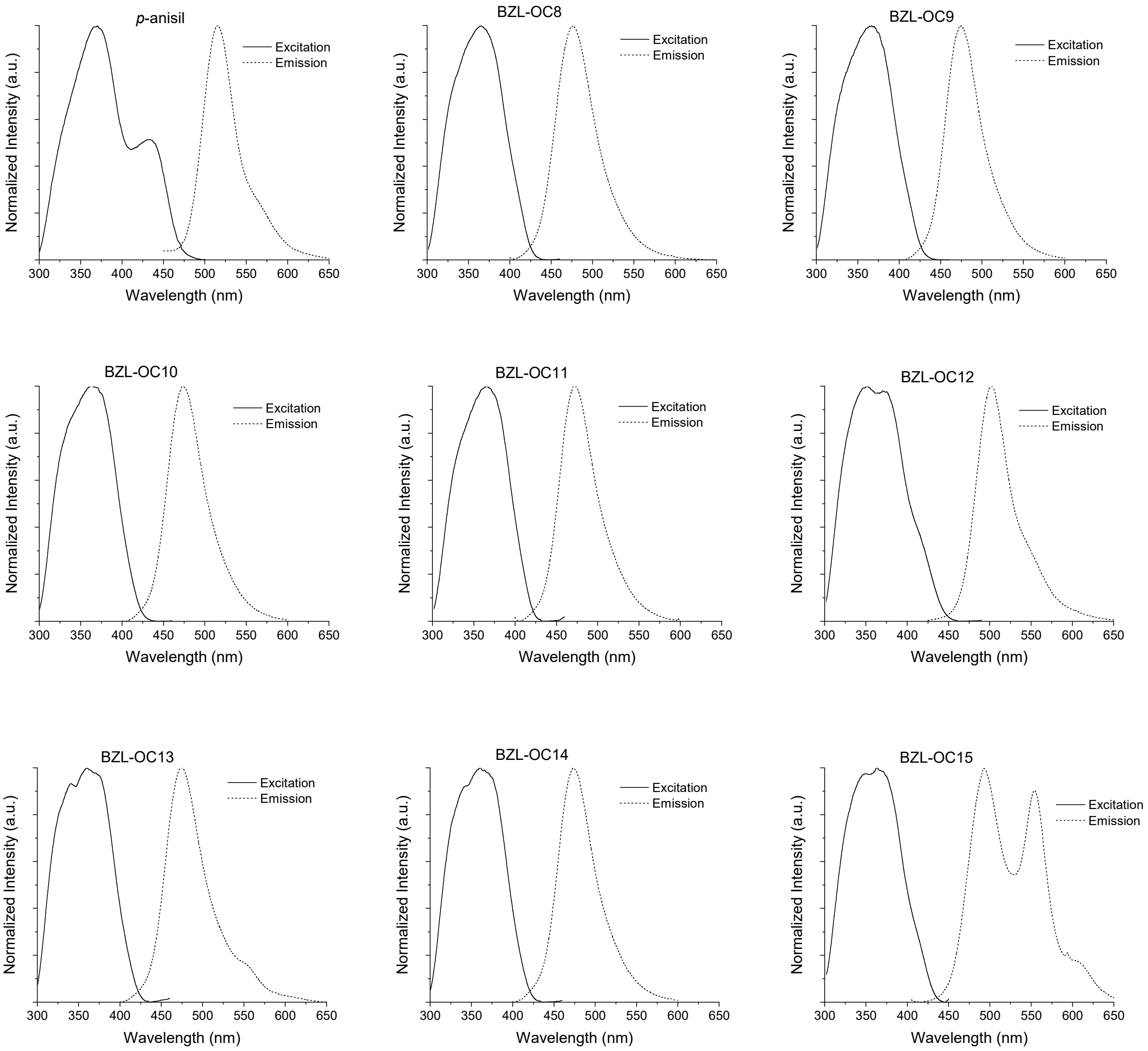

Figure S1. Excitation and emission spectra of *p*-anisil and BZL-OCn molecules in a crystalline form.

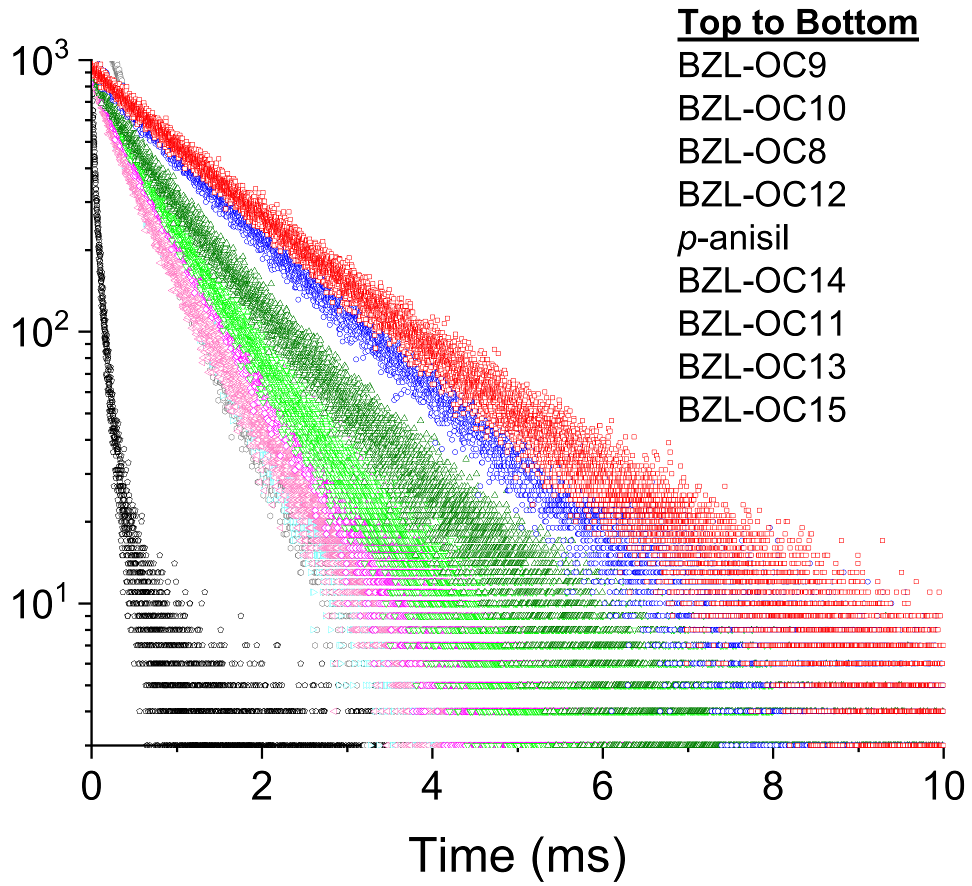

Figure S2. Time-resolved PL decay curves of BZL-OCn crystals and *p*-anisil crystal measured at their emission maxima.

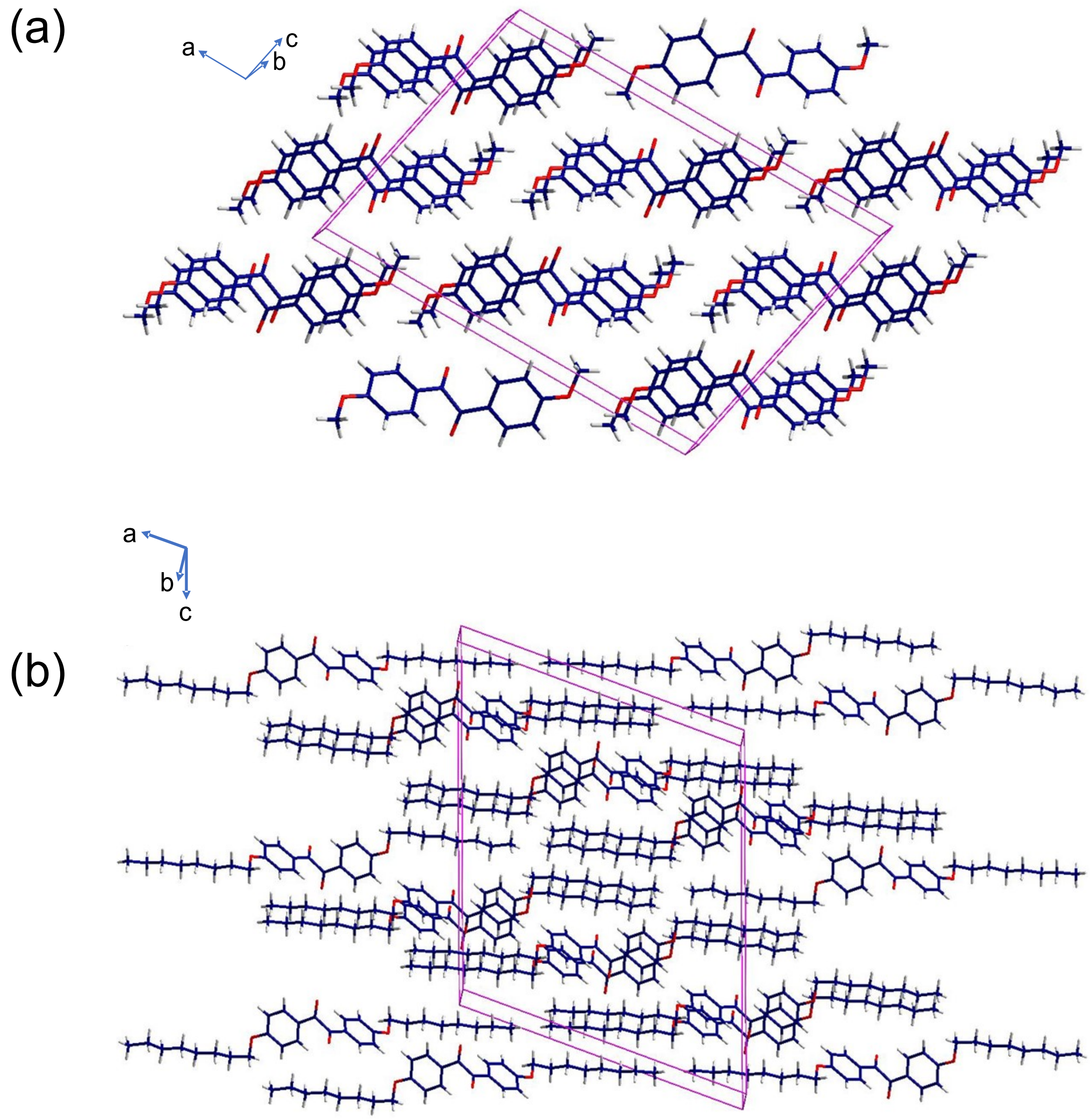

Figure S3. The crystal structure a) *p*-anisil and b) **BZL-OC8** with unit cell.

Table S1. Crystal data and structure refinement for *p*-anisil.

Empirical formula	$C_{16}H_{14}O_4$	
Formula weight	270.27	
Temperature	100(2) K	
Wavelength	0.71073 Å	
Crystal system	Monoclinic	
Space group	C2/c	
Unit cell dimensions	a = 22.042(5) Å	a= 90°
	b = 3.9504(9) Å	b= 104.448(3)°
	c = 15.024(3) Å	g = 90°
Volume	1266.8(5) $Å^3$	
Z	4	
Density (calculated)	1.417 Mg/m^3	
Absorption coefficient	0.102 mm^{-1}	
F(000)	568	
Theta range for data collection	1.908 to 30.507°	
Index ranges	-30<=h<=30, -5<=k<=5, -21<=l<=21	
Reflections collected	9695	
Independent reflections	1937 [R(int) = 0.0296]	
Completeness to theta = 25.242°	100.0 %	
Absorption correction	Semi-empirical from equivalents	
Max. and min. transmission	0.7466 and 0.7002	
Refinement method	Full-matrix least-squares on F^2	
Data / restraints / parameters	1937 / 0 / 92	
Goodness-of-fit on F^2	1.060	
Final R indices [I>2sigma(I)]	R_1 = 0.0434, wR_2 = 0.1209	
R indices (all data)	R_1 = 0.0476, wR_2 = 0.1262	
Extinction coefficient	n/a	
Largest diff. peak and hole	0.368 and -0.306 e.$Å^{-3}$	

Table S2. Crystal data and structure refinement for **BZL-OC8**.

Empirical formula	$C_{30}H_{42}O_4$	
Formula weight	466.63	
Temperature	100(2) K	
Wavelength	0.71073 Å	
Crystal system	Monoclinic	
Space group	C2/c	
Unit cell dimensions	a = 22.098(8) Å	a= 90°
	b = 4.7567(17) Å	b= 109.636(5)°
	c = 26.394(10) Å	g = 90°
Volume	2613.0(16) $Å^3$	
Z	4	
Density (calculated)	1.186 Mg/m^3	
Absorption coefficient	0.077 mm^{-1}	
F(000)	1016	
Theta range for data collection	1.638 to 26.372°	
Index ranges	-27<=h<=27, -5<=k<=5, -32<=l<=32	
Reflections collected	11158	
Independent reflections	2658 [R(int) = 0.0404]	
Completeness to theta = 25.242°	100.0 %	
Refinement method	Full-matrix least-squares on F^2	
Data / restraints / parameters	2658 / 0 / 155	
Goodness-of-fit on F^2	1.116	
Final R indices [I>2sigma(I)]	R_1 = 0.0472, wR_2 = 0.1230	
R indices (all data)	R_1 = 0.0631, wR_2 = 0.1332	
Extinction coefficient	n/a	
Largest diff. peak and hole	0.223 and -0.276 e.$Å^{-3}$	

References

1. G.M. Sheldrick, *Acta Cryst*., 2015, **A71**, 3-8.
2. G.M. Sheldrick, *Acta Cryst*., 2015, **C71**, 3-8.
3. O. V. Dolomanov, L. J. Bourhis, R. J. Gildea, J. A. K. Howard and H. Puschmann, *J. Appl. Cryst*. 2009, **42**, 339-341.
4. M. Jarvid, A. Johansson, J. M. Bjuggren, H. Wutzel, V. Englund, S. Gubanski, C. Müller and M. R. Andersson, *J. Polym. Sci. Part B: Polym. Phys.,* 2014, **52**, 1047-1054.
5. C. Yin, G. Wen, C. Liu, B. Yang, S. Lin, J. Huang, P. Zhao, S. H. D. Wong, K. Zhang, X. Chen, G. Li, X. Jiang, J. Huang, K. Pu, L. Wang and L. Bian, *ACS Nano,* 2018, **12**, 12201-12211.
6. K. Ohta, A. Takagi, H. Muroki, I. Yamamoto, K. Matsuzaki, T. Inabe and Y. Maruyama, *Mol. Cryst. Liq. Cryst.,* 1987, **147**, 15-24.
7. T.-T. Bui, O. Thiebaut, E. Grelet, M.-F. Achard, B. Garreau-de Bonneval and K. I. Moineau-Chane Ching, *Eur. J. Inorg. Chem.,* 2011, **17**, 2663-2676.
8. C.-T. Chou, Y. F. Pai, C.-C. Lin, T. K. Misra and C.-Y. Liu, *J. Chromatogr. A*., 2004, **1043**, 255-263.
9. T.-T. Bui, B. Garreau-de Bonneval and K. I. Moineau-Chane Ching, *New. J. Chem.,* 2010, **34**, 337-347.
10. H. Horie, A. Takagi, H. Hasebe, T. Ozawa and K. Ohta, *J. Mater. Chem*., 2001, **11**, 1063-1071.
